# Tongue Microbiota and Oral Health Status in Community-Dwelling Elderly Adults

**DOI:** 10.1128/mSphere.00332-18

**Published:** 2018-08-15

**Authors:** Mikari Asakawa, Toru Takeshita, Michiko Furuta, Shinya Kageyama, Kenji Takeuchi, Jun Hata, Toshiharu Ninomiya, Yoshihisa Yamashita

**Affiliations:** a Section of Preventive and Public Health Dentistry, Division of Oral Health, Growth and Development, Faculty of Dental Science, Kyushu University, Fukuoka, Japan; b OBT Research Center, Faculty of Dental Science, Kyushu University, Fukuoka, Japan; c Department of Epidemiology and Public Health, Graduate School of Medical Sciences, Kyushu University, Fukuoka, Japan; d Center for Cohort Studies, Graduate School of Medical Sciences, Kyushu University, Fukuoka, Japan; University of Wisconsin—Madison

**Keywords:** community-dwelling, dental, elderly, microbiota, oral, tongue

## Abstract

Tongue microbiota are a dominant source of oral microbial populations that are ingested with saliva, and therefore careful attention is required for the maintenance of health of elderly adults, who are susceptible to aspiration of oral contents. This study aimed to investigate the variation in tongue microbiota among community-dwelling elderly adults. Following a dental examination, tongue coating was collected from a 15-mm-diameter circular area at the center of the tongue dorsum of 506 elderly adults aged 70 to 80 years inhabiting the town of Hisayama, Japan. The microbial composition and density were determined by a 16S rRNA gene sequencing approach using a next-generation sequencer and quantitative PCR analysis, respectively. Co-occurrence network analysis identified two cohabiting groups of predominant commensals, one of which was primarily composed of Prevotella histicola, Veillonella atypica, Streptococcus salivarius, and Streptococcus parasanguinis; these organisms have been previously associated with an increased risk of mortality due to pneumonia in the frail elderly. This bacterial group was more predominant in the elderly with fewer teeth, a higher plaque index, and more dental caries experience, whereas the total bacterial density was independent of these traits. A higher density of fungi was also observed in the elderly with these traits, as well as in individuals who wore dentures. These results suggest that elderly adults with poorer oral health swallow a more dysbiotic microbiota formed on the tongue.

**IMPORTANCE** Aspiration of oral contents can lead to pneumonia, which is a major cause of death among elderly adults susceptible to swallowing impairments. Tongue microbiota are a dominant source of oral microbial populations that are ingested with saliva. This large-scale population-based study revealed variations in the tongue microbiota among community-dwelling elderly adults. The total bacterial density was independent of the conditions of teeth surrounding the tongue, whereas the microbiota composition, especially the relative abundances of predominant commensals, showed an association with tooth conditions. Our results demonstrate that the elderly with fewer teeth, poorer dental hygiene, and more dental caries experience constantly ingest more dysbiotic microbiota, which could be harmful for their respiratory health.

## Abstract (precis)

Aspiration of oral contents can lead to pneumonia, which is a major cause of death among elderly adults susceptible to swallowing impairments. Tongue microbiota are a dominant source of oral microbial populations that are ingested with saliva. This large-scale population-based study revealed variations in the tongue microbiota among community-dwelling elderly adults. The total bacterial density was independent of the conditions of teeth surrounding the tongue, whereas the microbiota composition, especially the relative abundances of predominant commensals, showed an association with tooth conditions. Our results demonstrate that the elderly with fewer teeth, poorer dental hygiene, and more dental caries experience constantly ingest more dysbiotic microbiota, which could be harmful for their respiratory health.

## INTRODUCTION

The human oral cavity is densely colonized by diverse microorganisms on the intraoral surfaces, which are constantly shed into saliva. Normally, the detached organisms ingested with saliva are transported via the esophagus into the stomach and are then inactivated by gastric acid and proteolytic enzymes (1). However, impairments in swallowing and cough reflux with aging allow for aspiration into the lower respiratory tract and subsequent pulmonary infection (2). Swallowing disorders are highly prevalent in elderly patients with pneumonia (3, 4), which is a major cause of death among elderly adults. Mechanical oral hygiene to decrease the oral microbial burden is recognized as an effective approach to reduce the mortality rate from aspiration pneumonia (5) and is introduced as standard nursing care to the frail elderly in the hospital and nursing home settings.

Although aspirated saliva contains microorganisms colonizing various oral sites, their bacterial composition indicates that the dominant source is the microbiota formed on the tongue (6–8). The dorsum of the tongue has a large surface area with papillary structures, which can retain numerous microorganisms, including both aerobes and anaerobes (9). The microbial community is packed less densely than dental plaque, allowing the bacterial cells ready access to sufficient nutrients via saliva (10). The loose community structure and the desquamation of epithelial cells facilitate the release of resident microorganisms into the saliva (11). These features imply that careful attention should be given to the tongue microbiota in the elderly susceptible to swallowing impairments (12). Furthermore, increased tongue coating in edentulous elderly adults, scored by visual inspections, was associated with aspiration pneumonia (13) and febrile status (14).

Using a 16S rRNA gene next-generation sequencing approach, we recently reported that the dysbiotic composition of tongue microbiota was also associated with a higher risk of death from pneumonia in frail elderly adults in nursing homes (15). This result suggests that methods to optimize the tongue microbiota composition might be beneficial in health maintenance of the elderly. However, no previous study has focused on the tongue microbiota composition of community-dwelling elderly adults, and therefore the tongue microbiota of normal elderly adults remains poorly understood. In this population-based study, we investigated the tongue microbiota and dental conditions of Japanese elderly adults aged 70 to 80 years inhabiting the town of Hisayama, Japan. This study aimed to understand the variations in tongue microbiota among community-dwelling elderly adults with various oral conditions and to identify oral health-related factors associated with the dysbiotic shift in the tongue microbiota.

## RESULTS

This study investigated the tongue microbiota status of 506 elderly adults aged 70 to 80 years (231 males and 275 females) inhabiting the town of Hisayama who received a dental examination during a health examination of Hisayama residents performed in 2016. Institutionalized or hospitalized elderly adults and very frail elderly residents, such as bedridden people, did not participate in the health examination or this study. Nevertheless, this study population accounts for 50.6% of the total residents of this town in this age group, suggesting that our data largely cover the variations in tongue microbiota among the independent elderly. The tongue microbiota was collected from the center area of the tongue dorsum using a modified electric toothbrush as the sampling device (see Fig. S1 in the supplemental material). The bacterial and fungal density per 15-mm-diameter circular area at the center of the tongue dorsum and the bacterial composition of the microbiota of each individual were investigated using quantitative PCR and 16S rRNA gene next-generation sequencing approaches, respectively.

10.1128/mSphere.00332-18.1 FIG S1  A tongue coating sampling device based on the Braun Oral-B Pro 500 electric toothbrush (Procter & Gamble, Cincinnati, OH) (A). A circular bonded-fiber fabric (15 mm in diameter) was attached to this brush head, from which the bristles were preliminarily removed (B). The head was placed on the center of the tongue dorsum, 3-s vibrations were applied, and the collected tongue coating samples adhered to the fabric. Download FIG S1, TIF file, 1.3 MB. Copyright © 2018 Asakawa et al. 2018 Asakawa et al. This content is distributed under the terms of the Creative Commons Attribution 4.0 International license.

### Tongue microbiota composition and predominant bacterial taxa.

The bacterial composition of tongue microbiota was determined by a 16S rRNA gene sequencing approach using a next-generation sequencer, Ion PGM (Thermo Fisher Scientific, Waltham, MA), which provided 9,544,681 quality-passed bacterial 16S rRNA gene sequences (V1-V2 regions). These sequences were assigned to 730 species-level operational taxonomic units (OTUs), using a cutoff distance of 0.03. Of these, 21 predominant OTUs with a mean relative abundance of >1% are listed in Table 1. The OTU corresponding to Streptococcocus salivarius indicated that this species was the most predominant in the tongue microbiota of the elderly, followed by other bacteria, such as Prevotella melaninogenica, Rothia mucilaginosa, and Veillonella atypica. The predominant taxa were mostly consistent with the Human Microbiome Project data, which recruited healthy younger people aged 18 to 40 years (16). These OTUs were mostly shared across this study population and constituted the majority of the microbiota in each individual (mean ± standard deviation [SD], 75.2% ± 9.5%).

**TABLE 1:** Twenty-one predominant OTUs with mean relative abundances of >1% in the tongue microbiota of 506 elderly adults

OTU no.	Bacterial species corresponding to each OTU (taxon ID)^a^	Mean relative abundance (%)	Detection rate (%)
OTU2	Streptococcus salivarius (755)	9.5 ± 8.6	99.4
OTU4	Prevotella melaninogenica (469)	9.2 ± 6.4	98.8
OTU1	Rothia mucilaginosa (681)	8.8 ± 8.1	99.6
OTU9	Veillonella atypica (524)	6.0 ± 3.2	100
OTU6	Neisseria flavescens (610)	5.8 ± 8.3	89.9
OTU3	Prevotella histicola (298)	4.7 ± 6.0	90.3
OTU19	Streptococcus parasanguinis II (411)	4.3 ± 3.9	98.0
OTU10	Actinomyces odontolyticus (701)	3.9 ± 3.4	99.6
OTU7	Granulicatella adiacens (534)	3.3 ± 2.5	99.6
OTU5	Haemophilus parainfluenzae (718)	3.2 ± 4.0	94.7
OTU421	Genus *Neisseria*^b^	2.4 ± 5.1	58.3
OTU25	*Actinomyces* sp. (172)	2.2 ± 2.9	93.5
OTU8	Fusobacterium periodonticum (201)	1.8 ± 2.3	90.5
OTU15	Prevotella pallens (714)	1.5 ± 1.5	91.3
OTU14	Porphyromonas pasteri (279)	1.5 ± 2.7	75.5
OTU11	Gemella sanguinis (757)	1.3 ± 1.3	97.2
OTU23	Actinomyces graevenitzii (866)	1.3 ± 2.1	88.1
OTU22	*Streptococcus* sp. (074)	1.3 ± 1.5	93.1
OTU28	Genus *Streptococcus*^b^	1.3 ± 1.5	96.0
OTU12	*Leptotrichia* sp. (417)	1.1 ± 2.8	86.8
OTU17	*Alloprevotella* sp. (308)	1.0 ± 1.4	85.2

a Oral taxon IDs in HOMD are given in parentheses following bacterial names.

b No BLAST hit with ≥98.5% identity was found in the Human Oral Microbiome Database (HOMD).

**FIG 1:**
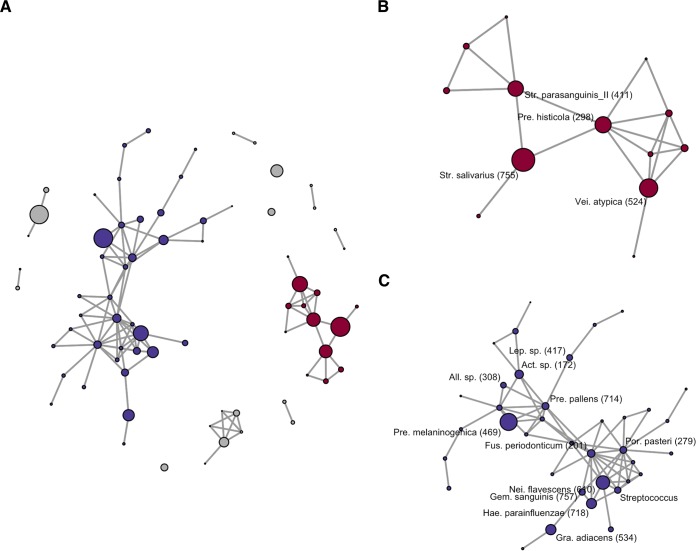
Co-occurrence network in tongue microbiota of 506 elderly adults built from SparCC correlation coefficients between sequence abundances. Nodes corresponding to operational taxonomic units (OTUs) and connecting edges indicate the correlation between them. Only correlations with values greater than 0.40 are represented as edges. The node size indicates a mean relative abundance of each OTU. (A) All OTUs excluding isolated nodes corresponding to the OTUs with a mean relative abundance of <1% are shown as nodes in the diagram (72 of all 730 OTUs). Most of the predominant OTUs (with mean relative abundance of >1%) belonged to either of the two major networks including multiple predominant OTUs (cohabiting commensal groups I and II). The nodes belonging to commensal groups I and II are colored red and blue, respectively. (B) Only the co-occurrence network of OTUs belonging to commensal group I is shown. The bacterial taxa corresponding to the predominant OTUs are described. Oral taxon IDs in the Human Oral Microbiome database are given in parentheses following the bacterial names. Abbreviations: Pre., *Prevotella*; Vei., *Veillonella*; Str., *Streptococcus*. (C) Here, only the co-occurrence network of OTUs belonging to commensal group II is shown. The bacterial taxa corresponding to the predominant OTUs are described. Oral taxon IDs in the Human Oral Microbiome database are given in parentheses following bacterial names. Abbreviations: Nei., *Neisseria*; Hae., *Haemophilus*; Por., *Porphyromonas*; Gra., *Granulicatella*; Fus., *Fusobacterium*; Pre., *Prevotella*; Act., *Actinomyces*; Lep., *Leptotorichia*; All., *Alloprevotella*.

**FIG 2:**
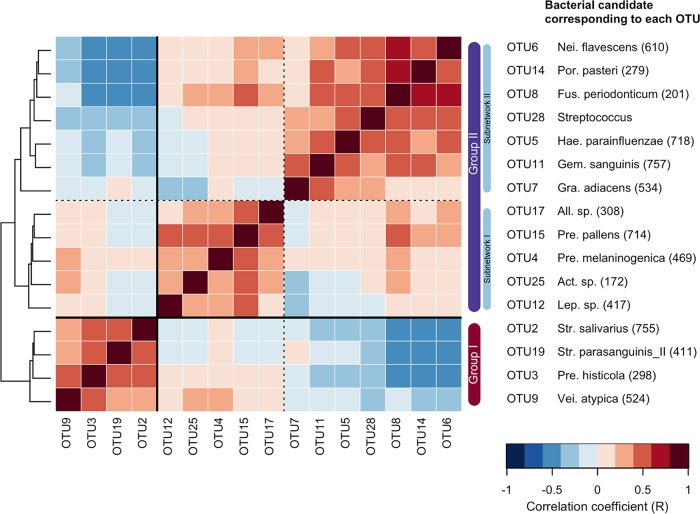
SparCC correlation coefficients between sequence abundances of predominant operational taxonomic units (OTUs) (with a mean relative abundance of >1%) belonging to cohabiting commensal groups I and II. The pairwise correlation coefficient is shown in each grid by the color intensity. The OTUs are ordered according to the result of a hierarchical cluster analysis using the Euclidean distance with average linkage (shown as a dendrogram on the left). Correlations with values greater than 0.40 are represented as edges in Fig. 1.

The co-occurrence network analysis using SparCC (17) suggested that these predominant members construct two major cohabiting groups (including multiple predominant OTUs) in the microbiota (Fig. 1A). One was primarily composed of a bacterial complex including Prevotella histicola, Veillonella atypica, Streptococcus salivarius, and Streptococcus parasanguinis II (commensal group I [Fig. 1B]). Another was mainly assembled from Neisseria flavescens, Haemophilus parainfluenzae, Fusobacterium periodonticum, Porphyromonas pasteri, Gemella sanguinis, Prevotella melaninogenica, and Prevotella pallens (commensal group II [Fig. 1C]). Negative associations were observed between the group I and group II bacteria, especially in subnetwork II (N. flavescens, F. periodonticum, P. pasteri, H. parainfluenzae, G. sanguinis, and Granulicatella adiacens [Fig. 1B and 2]). Principal-coordinate analysis (PCoA) plots based on unweighted UniFrac metrics showed that the individuals with microbiota containing greater relative abundances of group I commensals were localized in the negative direction of principal coordinate 1, whereas, in contrast, those with microbiota containing greater relative abundances of group II commensals were localized in its positive direction (see Fig. S2 in the supplemental material). It is suggested that the ratio of the two predominant commensal groups is strongly associated with the overall composition of the tongue microbiota.

10.1128/mSphere.00332-18.2 FIG S2  A principal-coordinate analysis plot showing similarity relationships among tongue microbiota samples from 506 community-dwelling elderly adults using an unweighted UniFrac distance metric. The points corresponding to the individuals with a higher relative abundance of each commensal group are depicted as bolder red. To correct for the unequal number of sequences, we evaluated 5,000 randomly selected sequences per sample. The two axes explained 12.9 and 9.7% of the variance, respectively. Download FIG S2, TIF file, 0.9 MB. Copyright © 2018 Asakawa et al. 2018 Asakawa et al. This content is distributed under the terms of the Creative Commons Attribution 4.0 International license.

Of the five predominant OTUs not belonging to the two commensal groups, the relative abundance of the OTU corresponding to Rothia mucilagiosa was significantly correlated with that of commensal group I, whereas those of the two OTUs corresponding to *Streptococcus* sp. strain OT-074 and *Neisseria* species were significantly correlated with commensal group II (see Table S1 in the supplemental material).

10.1128/mSphere.00332-18.5 TABLE S1  Spearman’s correlation coefficient between the relative abundances of group I and II commensals and five predominant operational taxonomic units (OTUs) that were not present in both cohabiting groups. Download TABLE S1, DOCX file, 0.1 MB. Copyright © 2018 Asakawa et al. 2018 Asakawa et al. This content is distributed under the terms of the Creative Commons Attribution 4.0 International license.

OTUs corresponding to mutans streptococci (MS) and lactobacilli, which are typical dental caries-associated taxa (18), and 10 periodontitis-associated taxa validated by Oliveira et al. (19) from 18 candidate bacteria listed in a systematic review (20) were detected from some of the individuals as minority members (mean relative abundance of <0.1% [Table 2]). Furthermore, they were not part of the above-mentioned cohabiting commensal groups.

**TABLE 2:** Relative abundances and detection rate of OTUs corresponding to mutans streptococci and lactobacilli and periodontitis-associated taxa in the tongue microbiota of 506 elderly adults

OTU no.	Bacterial species corresponding to each OTU (taxon ID)^a^	Mean relative abundance (%)	Detection rate (%)
14 OTUs corresponding to mutans streptococci and lactobacilli			
OTU73	Streptococcus mutans (686)	0.020 ± 0.131	17.8
OTU75	Streptococcus sobrinus (768)	0.039 ± 0.412	11.1
OTU157	Lactobacillus iners (838)	0.002 ± 0.055	0.2
OTU230	Lactobacillus paracasei (716)	0.001 ± 0.008	2.2
OTU235	Lactobacillus fermentum (608)	0.023 ± 0.222	4.9
OTU238	Lactobacillus vaginalis (051)	0.008 ± 0.064	4.0
OTU291	Lactobacillus ultunensis (461)	0.001 ± 0.011	3.0
OTU293	Lactobacillus reuteri (938)	0.001 ± 0.020	0.6
OTU338	Lactobacillus oris (709)	0.001 ± 0.007	1.2
OTU375	Lactobacillus pentosus (883)	0.000 ± 0.004	0.6
OTU40	Lactobacillus gasseri (615)	0.042 ± 0.437	8.3
OTU41	Lactobacillus crispatus (817)	0.029 ± 0.426	4.7
OTU47	Lactobacillus salivarius (756)	0.077 ± 0.722	8.5
OTU599	*Lactobacillus* sp. (052)	0.000 ± 0.001	0.2
11 OTUs corresponding to periodontitis-associated taxa^b^			
OTU60	Porphyromonas gingivalis (619)	0.066 ± 0.168	49.4
OTU92	Tannerella forsythia (613)	0.008 ± 0.021	22.3
OTU167	Filifactor alocis (539)	0.003 ± 0.012	8.3
OTU194	Selenomonas sputigena (151)	0.001 ± 0.008	4.0
OTU228	Selenomonas sputigena (151)	0.004 ± 0.023	10.9
OTU239	*Fretibacterium* sp. (360)	0.002 ± 0.007	6.5
OTU254	*Bacteroidales* sp. (274)	0.002 ± 0.011	7.1
OTU534	*Bacteroidales* sp. (274)	0.000 ± 0.002	0.8
OTU286	*Desulfobulbus* sp. (041)	0.000 ± 0.003	2.0
OTU346	*Fretibacterium* sp. (362)	0.001 ± 0.006	2.8
OTU81	TM7 sp. (356)	0.011 ± 0.031	21.7

a Oral taxon IDs in HOMD are given in parentheses following bacterial names.

b Bacterial taxa listed as periodontitis-associated bacteria by Oliveira et al. (19).

### Correlation between parameters associated with tongue microbiota status.

To investigate the relationship between tongue microbiota status and dental conditions, this study focused on total bacterial density and richness, total fungal density, and the relative abundances of the OTUs corresponding to four bacterial groups: MS and lactobacilli as typical dental caries-associated taxa (14 OTUs [Table 2]) (18), the above-mentioned 10 periodontitis-associated taxa listed in a previous study (11 OTUs [Table 2]) (19), and the predominant bacteria in commensal group I (4 OTUs [Fig. 1B and 2]) and II (12 OTUs [Fig. 1C and 2]).

Prior to this analysis, we evaluated the relationships between eight parameters associated with the microbiota. Spearman’s correlation coefficients for these parameters are described in Table 3. A tongue coating score based on visual inspections of the tongue coating area and thickness reflected the total bacterial density rather than the bacterial composition. A higher relative abundance of periodontitis-associated taxa showed a significant association with a greater bacterial richness of the microbiota (number of observed OTUs). This relationship is reasonable, considering that deepening periodontal pockets resulting from periodontitis progression supply saliva to the subgingival site-specific bacteria, which are rarely present on the tongue. Of the two cohabiting commensal groups, the relative abundance of group I was positively correlated with the relative abundance of the dental caries-associated bacterial group (MS and lactobacilli) and the fungal density. On the other hand, their relative abundances exhibited inverse correlations with the relative abundances of the members of commensal group II.

**TABLE 3:**
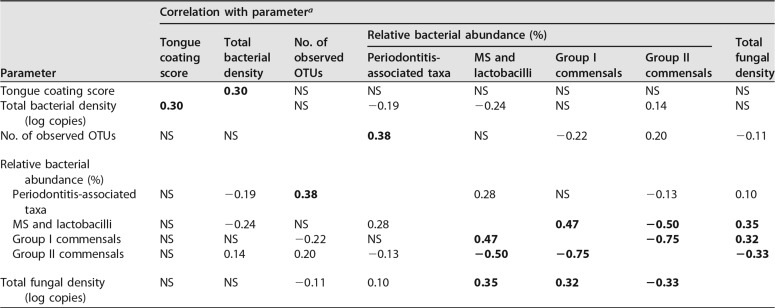
Spearman’s correlation coefficient between parameters associated with the tongue microbiota status

Parameter	Correlation with parameter^a^							
	Tongue coating score	Total bacterial density	No. of observed OTUs	Relative bacterial abundance (%)				Total fungal density
				Periodontitis- associated taxa	MS and lactobacilli	Group I commensals	Group II commensals	
Tongue coating score		**0.30**	NS	NS	NS	NS	NS	NS
Total bacterial density (log copies)	**0.30**		NS	−0.19	−0.24	NS	0.14	NS
No. of observed OTUs	NS	NS		**0.38**	NS	−0.22	0.20	−0.11
Relative bacterial abundance (%)								
Periodontitis-associated taxa	NS	−0.19	**0.38**		0.28	NS	−0.13	0.10
MS and lactobacilli	NS	−0.24	NS	0.28		**0.47**	**−0.50**	**0.35**
Group I commensals	NS	NS	−0.22	NS	**0.47**		**−0.75**	**0.32**
Group II commensals	NS	0.14	0.20	−0.13	**−0.50**	**−0.75**		**−0.33**
Total fungal density (log copies)	NS	NS	−0.11	0.10	**0.35**	**0.32**	**−0.33**	

a An absolute correlation coefficient of >0.3 is shown in boldface. NS, not significant.

### Tongue microbiota status and oral health conditions.

**FIG 3:**
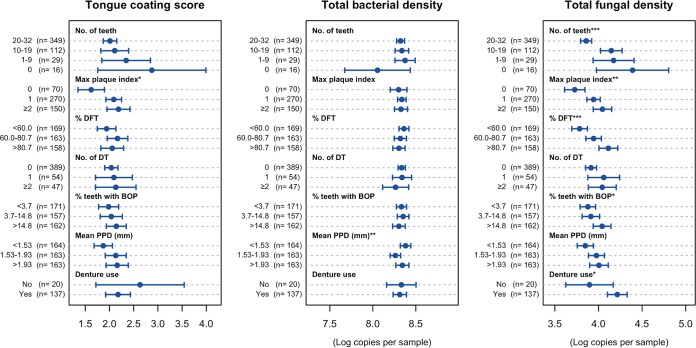
Tongue coating score and total bacterial and fungal density per 15-mm-diameter circular area at the center of the tongue dorsum of the elderly adults with various oral conditions. Of the 506 individuals, the 16 edentulous elderly were excluded from the maximum plaque index, percentage of decayed and filled teeth (DFT), number of decayed teeth (DT), percentage of teeth with bleeding on probing (BOP), and mean periodontal pocket depth (PPD), whereas 349 individuals with ≥20 teeth were excluded from denture use. The individuals were categorized into the tertile of percentage of DFT, percentage of teeth with BOP, and mean PPD, and then the parameters of the microbiota status were compared. Dots indicate the mean; the error bars indicate 95% confidence intervals. ***, *P* < 0.001, **, *P* < 0.01, and *, *P* < 0.05, by Kruskal-Wallis analysis.

**FIG 4:**
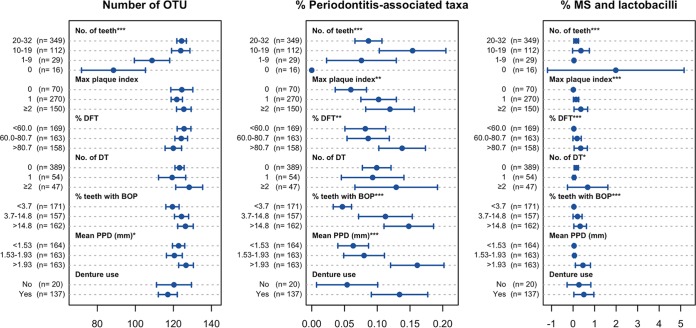
Alpha diversity and relative abundances of periodontitis- and dental caries-associated taxa in tongue microbiota of the elderly adults with various oral conditions. Of the 506 individuals, the 16 edentulous elderly were excluded from the maximum plaque index, percentage of decayed and filled teeth (DFT), number of decayed teeth (DT), percentage of teeth with bleeding on probing (BOP), and mean periodontal pocket depth (PPD), whereas 349 participants with ≥20 teeth were excluded from denture use. The individuals were categorized into the tertile of percentage of DFT, percentage of teeth with BOP, and mean PPD, and the parameters of the microbiota status were compared. Dots indicate the mean; the error bars indicate 95% confidence intervals. ***, *P* < 0.001, **, *P* < 0.01, and *, *P* < 0.05, by Kruskal-Wallis analysis.

**FIG 5:**
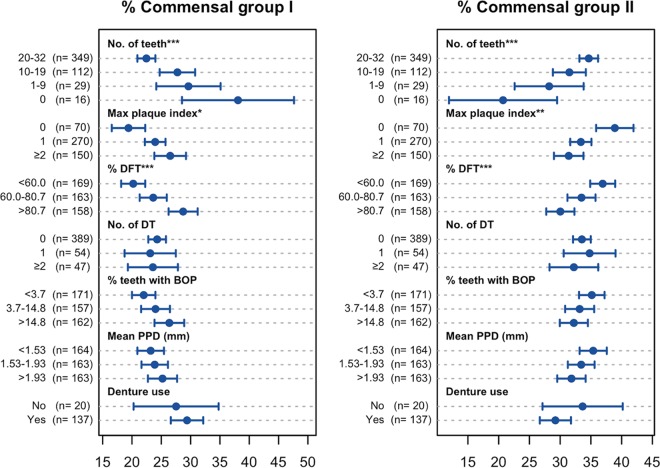
Relative abundances of two cohabiting commensal groups in tongue microbiota of elderly adults with various oral conditions. Of the 506 individuals, the 16 edentulous elderly were excluded from the maximum plaque index, percentage of decayed and filled teeth (DFT), number of decayed teeth (DT), percentage of teeth with bleeding on probing (BOP), and mean periodontal pocket depth (PPD), whereas 349 participants with ≥20 teeth were excluded from denture use. The individuals were categorized into the tertile of percentage of DFT, percentage of teeth with BOP, and mean PPD, and the parameters of the microbiota status were compared. Dots indicate the mean; the error bars indicate 95% confidence intervals. ***, *P* < 0.001, **, *P* < 0.01, and *, *P* < 0.05, in Kruskal-Wallis analysis.

We compared the above-mentioned tongue microbiota statuses among the elderly with various oral conditions, including the number of remaining teeth, dental hygiene (maximum plaque index), dental caries experience (percentage of caries-experienced teeth), active dental caries (number of decayed teeth), gingivitis (percentage of teeth with bleeding on probing), periodontitis (mean periodontal pocket depth), and the use of dentures (Fig. 3, 4, and 5).

A notable difference was not observed in the tongue coating scores based on visual inspections, except for a low score in the individuals without visible dental plaque (Fig. 3). The bacterial density of the tongue microbiota was also mostly independent of the oral conditions (Fig. 3). Although a significant difference in bacterial densities was observed among the individuals with different mean periodontal pocket depths, the density in the middle category of mean periodontal pocket depth was lower than in the other categories. The cause of this nonlinear relationship remains unclear.

A higher density of fungi was observed in the microbiota of individuals with less than 20 teeth, a higher plaque index, and more caries-experienced teeth (Fig. 3). In the individuals with less than 20 teeth, the use of dentures was significantly associated with a higher fungal density.

A drastic decrease in the bacterial richness (number of observed OTUs) was observed in the individuals with fewer teeth (Fig. 4). This reduction was mostly due to the dissipation of various minority taxa in the tongue microbiota, rather than the elimination of specific predominant members. It is likely to be caused by the supply decline of bacteria shed from tooth surfaces.

Periodontitis-associated taxa were enriched in the individuals with a greater amount of dental plaque and gingival and periodontal inflammation, as expected (Fig. 4). The lack of periodontitis-associated taxa in the edentulous individuals was not unexpected, as the periodontal niches disappeared from the oral cavity due to the loss of the tooth itself. A higher abundance of MS and lactobacilli was observed in the individuals with multiple decayed teeth, more caries-experienced teeth, and a larger amount of dental plaque, as well as those with more severe gingivitis and edentulous individuals (Fig. 4).

The ratio of the two predominant commensal groups varied drastically in accordance with tooth conditions and dental hygiene (Fig. 5). Commensal group I, including P. histicola, was observed in a higher proportion in the microbiota of individuals with less teeth, a higher plaque index, and more caries-experienced teeth. On the other hand, their microbiota contained a lower abundance of group II commensals such as N. flavescens.

The PCoA plot based on unweighted UniFrac metrics showed that the individuals with better oral health status tend to localize in the positive direction of principal coordinate 1 (see Fig. S3 in the supplemental material). The significant relationships between overall composition of the tongue microbiota and five oral health-related parameters (the number of remaining teeth, maximum plaque index, percentage of caries-experienced teeth, percentage of teeth with bleeding and on probing, and mean periodontal pocket depth) were confirmed by permutational analysis of variance (PERMANOVA) (Table 4).

10.1128/mSphere.00332-18.3 FIG S3  A principal-coordinate analysis plot showing similarity relationship among tongue microbiota samples from 506 community-dwelling elderly adults using an unweighted UniFrac distance metric. The points corresponding to the individuals with different categories in each oral health status are depicted in different colors. To correct for the unequal number of sequences, we evaluated 5,000 randomly selected sequences per sample. The two axes explained 12.9 and 9.7% of the variance, respectively. Download FIG S3, TIF file, 1.4 MB. Copyright © 2018 Asakawa et al. 2018 Asakawa et al. This content is distributed under the terms of the Creative Commons Attribution 4.0 International license.

**TABLE 4:** PERMANOVA results for variance of the unweighted UniFrac distance among 490 dentulous elderly adults

Variable	*R*^2^ value	*P* value
Gender	0.002	0.631
No. of teeth (3 categories)^a,b^	0.011	<0.001
Max plaque index (3 categories)^a^	0.009	<0.001
% DFT (3 categories)^a^	0.009	<0.001
No. of DT (3 categories)^a^	0.004	0.290
% of teeth with BOP (3 categories)^a^	0.008	<0.001
Mean PPD (mm [3 categories])^a^	0.007	<0.001
Denture use	0.002	0.197
Residuals	0.944	
Total	1.000	

a The category definition of each oral health status is described in Fig. 3, 4, and 5.

b The category including edentulous individuals was excluded from this analysis.

## DISCUSSION

This population-based study revealed the tongue microbiota composition of 506 Japanese community-dwelling elderly adults aged 70 to 80 years. The predominant bacterial taxa, including S. salivarius, were mostly common among the individuals (Table 1). On the other hand, a co-occurrence network analysis suggested that these commensal members constructed two competitive cohabiting groups in the microbiota (Fig. 1 and 2), and the ratio of these two groups varied among individuals with different dental conditions (Fig. 5). One group assembled from group I commensals, including N. flavescens and P. pasteri, was observed in a lower proportion in the microbiota of individuals with fewer teeth, poorer dental hygiene, and more dental caries experience. Alternatively, the other group composed of group II commensals, including P. histicola, V. atypica, S. salivarius, and S. parasanguinis, was more predominant in their microbiota. These bacteria were specifically predominant in the microbiota on oral mucosal surfaces, including the tongue dorsum, and were only minor components of the tooth-associated microbiota (16, 21). Therefore, there was little doubt that the observed differences occurred in the tongue microbiota itself, rather than from the contamination of dental plaque shed from the teeth. Our result suggests that the global composition of the tongue microbiota was associated with the tooth conditions surrounding but separated from the tongue, although whether it is the cause or consequence of dental conditions remains unclear.

The tongue microbiota is a primary source of numerous bacterial populations ingested with saliva (6–8). The results of this study imply that the laryngopharynx of the elderly with fewer teeth, poorer dental hygiene, and more dental caries experience is constantly exposed to bacterial populations containing a higher relative abundance of group I commensals. Although *Prevotella*, *Veillonella*, and *Streptococcus* are not typical respiratory pathogens, they were identified as etiologic agents of pulmonary infections such as aspiration pneumonia (22) and lung abscesses (23). Our prospective cohort study demonstrated that a higher relative abundance of group I commensals on the tongue was implicated in an increased mortality risk from pneumonia in frail elderly adults in nursing homes (15). It is assumed that an aspiration of oral contents into the lower respiratory tract would be more health threatening for the elderly with poorer dental conditions. In addition, recent studies revealed that the lungs of healthy adults are colonized by bacteria mainly carried by microaspiration, which frequently contains *Prevotella*, *Veillonella*, and *Streptococcus* species (24). Segal et al. also demonstrated that the predominance of *Prevotella* and *Veillonella* species in the lung microbiota was associated with enhanced subclinical lung inflammation, such as enhanced expression of inflammatory cytokines and elevated Th17 lymphocytes (25). These results suggest that the tongue microbiota in which group I commensals are predominant would also be undesirable in the nondysphagic elderly.

The relative abundance of group I commensals was significantly correlated with that of MS and lactobacilli, which are well-known aciduric bacteria associated with dental caries (Table 3). The commensal group I itself also included the two predominant *Streptococcus* species on the tongue, i.e., S. salivarius and S. parasanguinis, which can survive in acidic conditions (26, 27), indicating an enrichment of the acid-tolerant bacteria in the tongue microbiota. In addition, elevated levels of the *Veillonella* species that metabolize lactate were associated with higher acid production within plaque (28). Therefore, it is reasonable to assume that the more acidified oral ecosystem is correlated with a tongue microbiota containing a predominance of group II commensals. The prolonged low-pH conditions might contribute to a higher susceptibility to dental caries, resulting in a larger number of dental caries-experienced teeth in the oral cavity.

A previous Dutch study demonstrated that a higher *Candida* load in the saliva of elderly adults occurred in aciduric bacterial communities with a greater prevalence of streptococci (29). Consistent with these results, a greater amount of fungi was contained in the tongue microbiota of the elderly with fewer teeth, poorer dental hygiene, and more dental caries experience, which contained higher relative abundances of *Streptococcus* species (Fig. 5). Synergistic interactions between streptococci and *Candida* via coaggregation, signaling, and metabolic processes (30) might promote their growth on the tongue. Furthermore, a previous study showed that the number of *Candida* species on the tongue dorsum tended to be higher in those subjects who died of aspiration pneumonia (31), although *Candida* pneumonia is a rare infection of the lung (32). The tongue dorsum should be treated with caution as a potential reservoir of oral fungi in the individuals who wore dentures (33), as well as in the dentate elderly with fewer teeth, poorer dental hygiene, and more dental caries experience.

No significant differences were observed in the relative abundance of both commensal groups I and II among individuals with different gingivitis (percentage of teeth with bleeding on probing) and periodontitis (mean periodontal pocket depth) statuses (Fig. 5), suggesting that the ratio of predominant commensals in the tongue microbiota was associated more strongly with tooth conditions compared with gingival conditions. Nevertheless, the tongue microbiota of elderly adults with poorer gingival or periodontal health contained a drastically higher relative abundance of the OTUs corresponding to periodontitis-associated taxa (Fig. 4), although they were only minor components even in the elderly with poor gingival health (up to 2.1%). Tongue microbiota analysis using a 16S rRNA gene deep sequencing approach would be useful for monitoring subgingival bacteria shed from the periodontal pockets.

The relative abundance of group I commensals varied with the tooth conditions of each elderly subject (Fig. 5); however, no significant relationship was observed with the total bacterial density on their tongue dorsum (Table 3). This result indicates that the predominance of group I commensals was unlikely to be a consequence of bacterial overgrowth on the tongue. Mechanical cleaning of the coated tongue is an effective approach to decrease the bacterial burden on the tongue dorsum (12, 34, 35). However, our data suggest that a future prospective intervention study is essential to develop another tongue care approach, which can systematically alter the ratio of the two commensal groups in the microbiota. Noteworthy is the inverse correlation between the group I and group II commensals in subnetwork II (N. flavescens, F. periodonticum, P. pasteri, H. parainfluenzae, G. sanguinis, and G. adiacens [Fig. 2]). Their growth promotion might be also helpful for shifting the microbiota to a healthier one.

Our previous population-based study demonstrated that saliva of both middle-aged and elderly adults often contains high relative abundances of group I commensals such as P. histicola, V. atypica, S. salivarius, and S. parasanguinis (36), suggesting that the dysbiotic pattern of the tongue microbiota is not unique to elderly adults. On the other hand, the study also indicated that the percentage of individuals with higher relative abundances of these taxa significantly increased according to age after adjustment for confounding factors (36). It is therefore assumed that dysbiotic tongue microbiota would occur with a higher frequency in older people.

Our large-scale population-based study revealed variations of the tongue microbiota among Japanese community-dwelling elderly adults aged 70 to 80 years. This result suggests that the elderly with less teeth, poorer dental hygiene, and more dental caries experience constantly ingest more dysbiotic microbiota, with a higher relative abundance of *Prevotella*, *Veillonella*, and *Streptococcus* species and a larger number of fungal species. Careful attention should be given to the tongue microbiota status in elderly adults with poorer dental conditions.

## MATERIALS AND METHODS

### Ethics approval and consent to participate.

All participants understood the nature of the study and provided informed consent. The Ethics Committee of Kyushu University, Fukuoka, Japan, approved the study design as well as the procedure for obtaining informed consent (reference no. 28-31). All experiments were performed in accordance with the approved guidelines.

### Study population.

Dental examinations and the collection of tongue coating samples were conducted for elderly residents in the town of Hisayama, Fukuoka, Japan, which is a suburb of the Fukuoka metropolitan area in western Japan. Hisayama is recognized to be demographically representative of Japan in terms of its age and occupational distributions based on national consensus data (37). The population of Hisayama is approximately 8,000. A health examination of adult residents, including dental examinations, was conducted in 2016. Among all residents aged 70 to 80 years (1,000 individuals), 508 residents who received the dental examination consented to participate in the study.

### Dental examination.

The details of the dental examination as a component of a health examination of Hisayama residents were described previously (36). In brief, the numbers of present, decayed, or filled teeth were examined. Periodontal conditions were evaluated based on periodontal pocket depth and bleeding on probing at two sites for all teeth (mesio- and mid-buccal sites) based on the NHANES III method, with the exception of the third molars. The tongue coating was visually scored by multiplying the area score (0 to 3) and thickness score (0 to 2) based on conventional criteria (38).

### Sample collection.

Following the dental examination, tongue coating samples were collected by using a sampling device based on a modified Braun Oral-B Pro 500 electric toothbrush (Procter & Gamble, Cincinnati, OH [Fig. S1]). A circular bonded-fiber fabric (15 mm in diameter; Kao Corporation, Tokyo, Japan) was attached to this brush head, of which the bristles were preliminarily removed, as described previously (39). The head was placed on the center of the tongue dorsum, 3-s vibrations were applied, and the collected tongue coating samples adhered to the fabric. The fabric peeled from the brush head was immersed in 200 µl of lysis buffer containing 10 mM Tris-HCl, 1 mM EDTA, and 1% sodium dodecyl sulfate and then stored at −80°C until further analysis. DNA extraction from the tongue coating sample was performed using a bead-beating method with 0.3 g zirconia silica beads (0.1-mm diameter [BioSpec Products, Bartlesville, OK]) and a tungsten-carbide bead (3-mm diameter [Qiagen, Hilden, Germany]) as described previously (40). Two individuals were excluded from the latter analysis due to the poor quality of the extracted DNA.

### Quantitative PCR analysis.

Quantitative PCR analysis of bacterial or fungal density in the collected sample was performed using a QuantiFast SYBR green PCR kit (Qiagen) according to the manufacturer’s instructions. The primers 806F (5′ TTA GAT ACC CYG GTA GTC C 3′) and 926R (5′ CCG TCA ATT YCT TTG AGT TT 3′), which target V5 regions of the 16S rRNA gene, were used for the quantification of bacterial density (41). The 16S rRNA gene of P. pasteri was inserted into the vector plasmid pBluescript SKII(+) (Stratagene, La Jolla, CA) and used as a real-time control. The fungal density was estimated using the primers 5.8SR (5′ TCG ATG AAG AAC GCA GC 3′) (42) and ITS4 (5′ TCC TCC GCT TAT TGA TAT GC 3′) (43) for the amplification of a fungal internal transcribed spacer 2 region of the rRNA operon. This primer set covers a variety of fungal species, excluding several species of basidiomycetous fungi (the phylum Basidiomycota) (44). The internal transcribed spacer region of Candida albicans was inserted into the vector plasmid pBluescript SKII(+) (Stratagene) and used as a real-time control.

### Ion Torrent 16S rRNA gene analysis.

The 16S rRNA gene sequencing analysis was performed using tongue coating samples collected from the participants. The V1-V2 region of 16S rRNA genes from each sample was amplified using the following primers: 8F (5′ AGA GTT TGA TYM TGG CTC AG 3′), with Ion Torrent adaptor A and the sample-specific 8-base tag sequence, and 338R (5′ TGC TGC CTC CCG TAG GAG T 3′>), with the Ion Torrent trP1 adaptor sequence. PCR amplification, purification, and quantification of each PCR adaptor were performed as previously described (36). Emulsion PCR and enrichment of template-positive particles were performed using an Ion PGM Hi-Q View OT2 kit (Thermo Fisher Scientific) with the Ion One Touch 2 system (Thermo Fisher Scientific), and sequencing was performed with the Ion PGM (Thermo Fisher Scientific) using an Ion PGM Hi-Q View sequencing kit (Thermo Fisher Scientific).

### Data processing.

The raw sequence reads were quality filtered using a script written in R (version 3.3.1). The reads were excluded from the analysis if they exhibited ≤240 bases (not including the tag sequence), had an average quality score of ≤25, did not include the correct forward primer sequence, did not include the correct reverse primer sequence (one mismatch was allowed), or had a homopolymer run of >7 nucleotides (nt). The remaining reads were assigned to the appropriate sample by examining the tag sequence. Similar sequences were assigned into operational taxonomic units (OTUs) using UPARSE (45), with a minimum pairwise identity of 97%. The taxonomy of representative sequences was determined using BLAST against 889 oral bacterial 16S rRNA gene sequences (HOMD 16S rRNA RefSeq version 14.51) in the Human Oral Microbiome Database (46). Nearest-neighbor species with ≥98.5% identity to the representative sequence were selected as candidates for each OTU. The taxonomy of sequences without hits was further determined using the RDP classifier with a minimum support threshold of 80%. The number of observed OTUs and the relative abundance of each OTU were calculated following rarefaction with a depth of 5,000 reads per sample using R. We confirmed that the rarefaction curve for the number of OTUs per sample approached a plateau after 5,000 sequence reads (see Fig. S4 in the supplemental material).

10.1128/mSphere.00332-18.4 FIG S4  Rarefaction curves for a number of observed operational taxonomic units (OTUs) per sample. The mean number of observed OTUs almost reached a plateau by 5,000 sequence reads. The error bars indicate the standard deviation. Download FIG S4, TIF file, 0.6 MB. Copyright © 2018 Asakawa et al. 2018 Asakawa et al. This content is distributed under the terms of the Creative Commons Attribution 4.0 International license.

### Statistical analysis.

All statistical analyses were conducted using R (version 3.3.1). The co-occurrence network analysis was conducted using SparCC (17), which calculates correlations from compositional data using a log-ratio-transformed abundance with default parameters (10 inference iterations and 100 bootstraps). Co-occurrence networks with correlation values greater than 0.40 were represented as edges and were constructed using the gplot function in the sna library. The unweighted UniFrac metric (47) was used to determine the dissimilarity between any pair of bacterial communities. The similarity relationship, assessed using the UniFrac metric, was presented in a principal-coordinate analysis (PCoA) plot drawn by R. Permutational multivariate analysis of variance (PERMANOVA) was used to assess the effects of oral health-related factors on tongue microbiota using the Adonis function in the vegan library based on 9,999 permutations. The tongue microbiota statuses were compared among the elderly with different dental and gingival conditions, which included the number of present teeth, dental hygiene (maximum plaque index), dental caries (number of decayed teeth or caries-experienced teeth), gingivitis (percentage of teeth with bleeding on probing), periodontitis (mean periodontal pocket depth), and the use of dentures. The conditions of dental hygiene and dental caries, as well as gingival and periodontal inflammation, were compared among the individuals with ≥1 tooth, because such parameters were unable to be evaluated for the edentulous individuals. Kruskal-Wallis analysis was used to assess the differences in microbiota status between the categories.

### Accession number(s).

The obtained sequence data obtained in this study have been deposited in the DDBJ Sequence Read Archive under accession no. DRA006979, DRA006980, DRA006981, DRA006982, and DRA006983.
