# Supplementary figures and images for: Tongue Microbiota and Oral Health Status in Community-Dwelling Elderly Adults
Source: mSphere. 2018 Aug 15;3(4):e00332-18. doi: 10.1128/mSphere.00332-18 (PMC6094060; doi:10.1128/mSphere.00332-18)

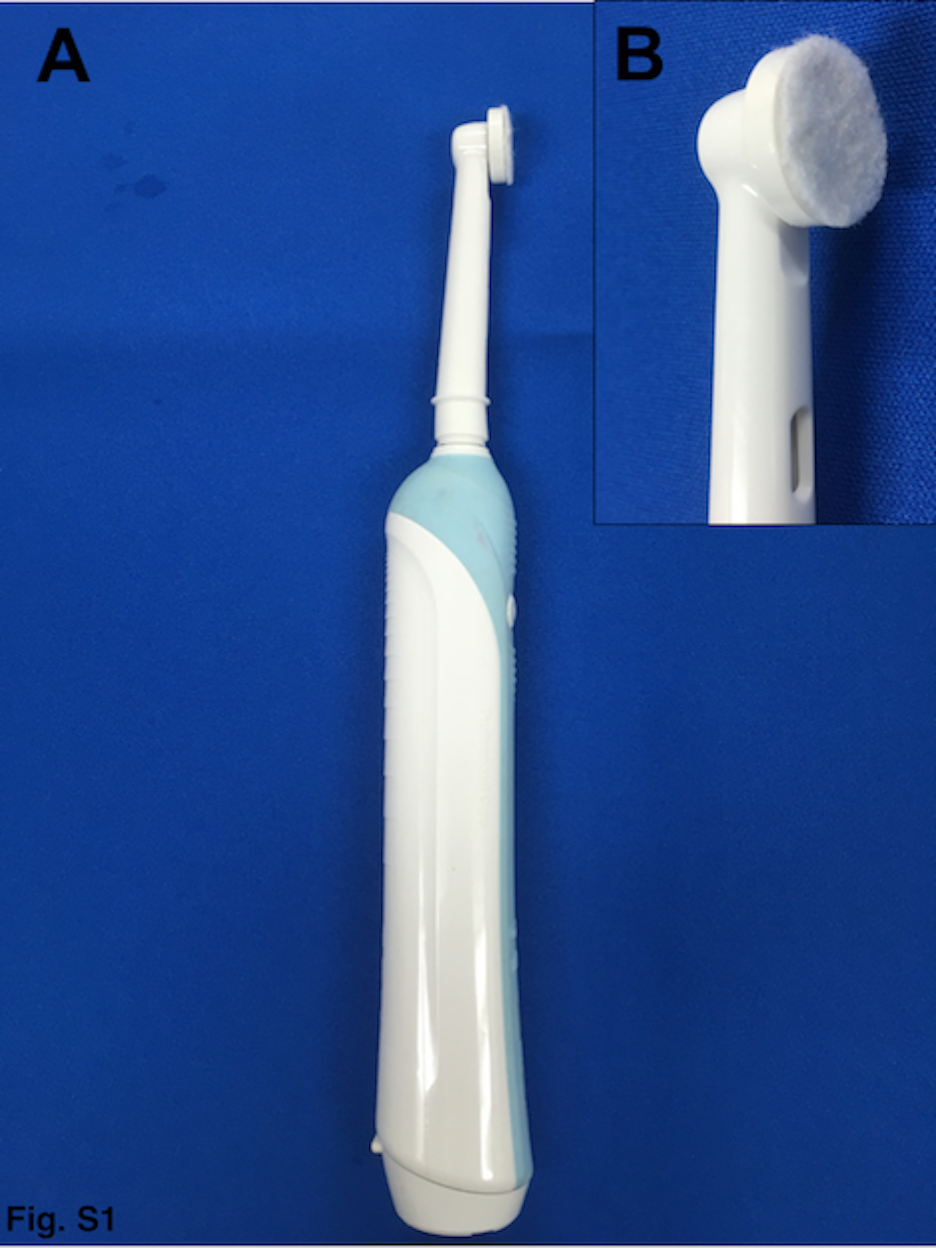

Supplement: FIG S1 [file sph004182614sf1.tif]

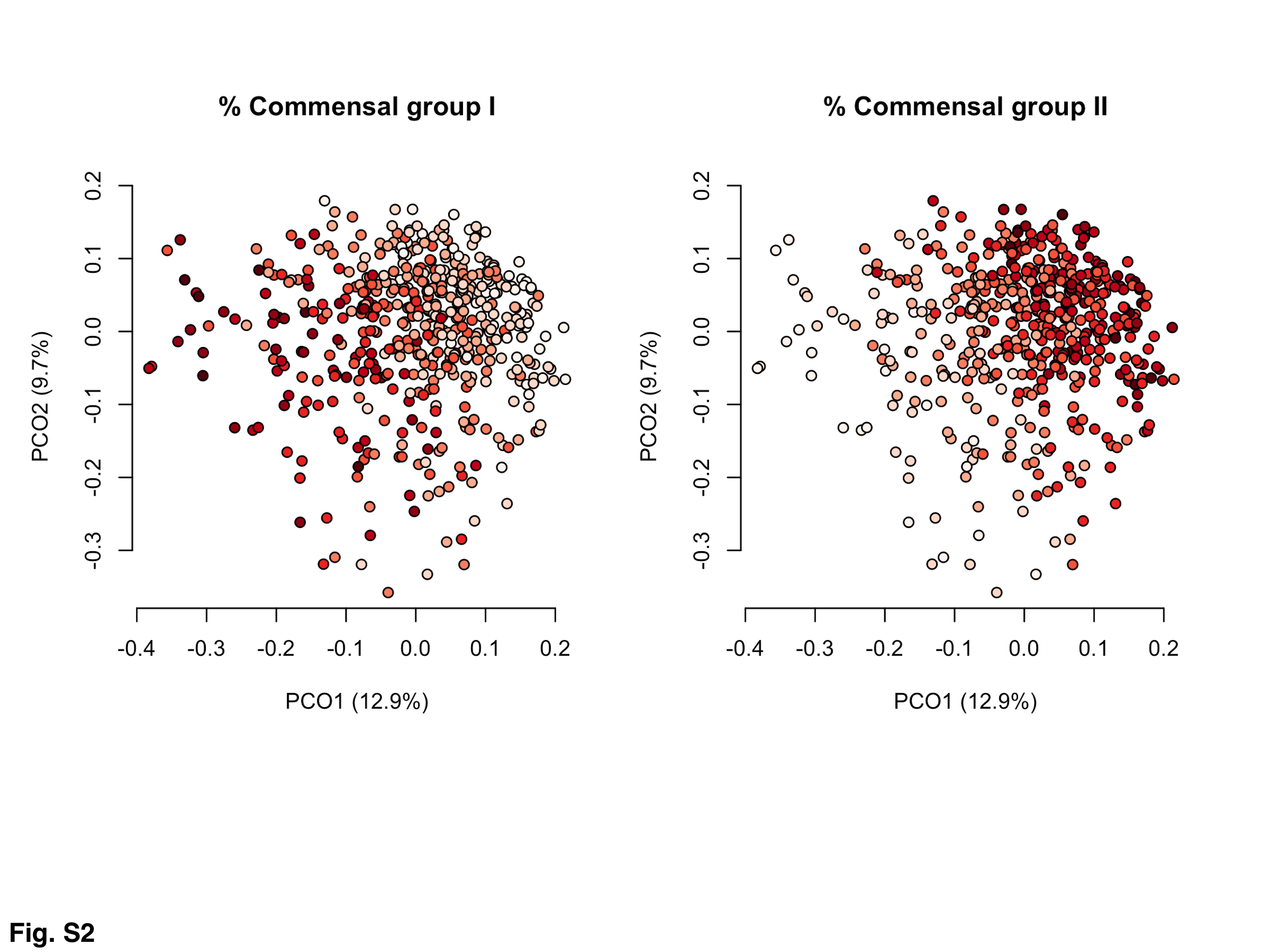

Supplement: FIG S2 [file sph004182614sf2.tif]

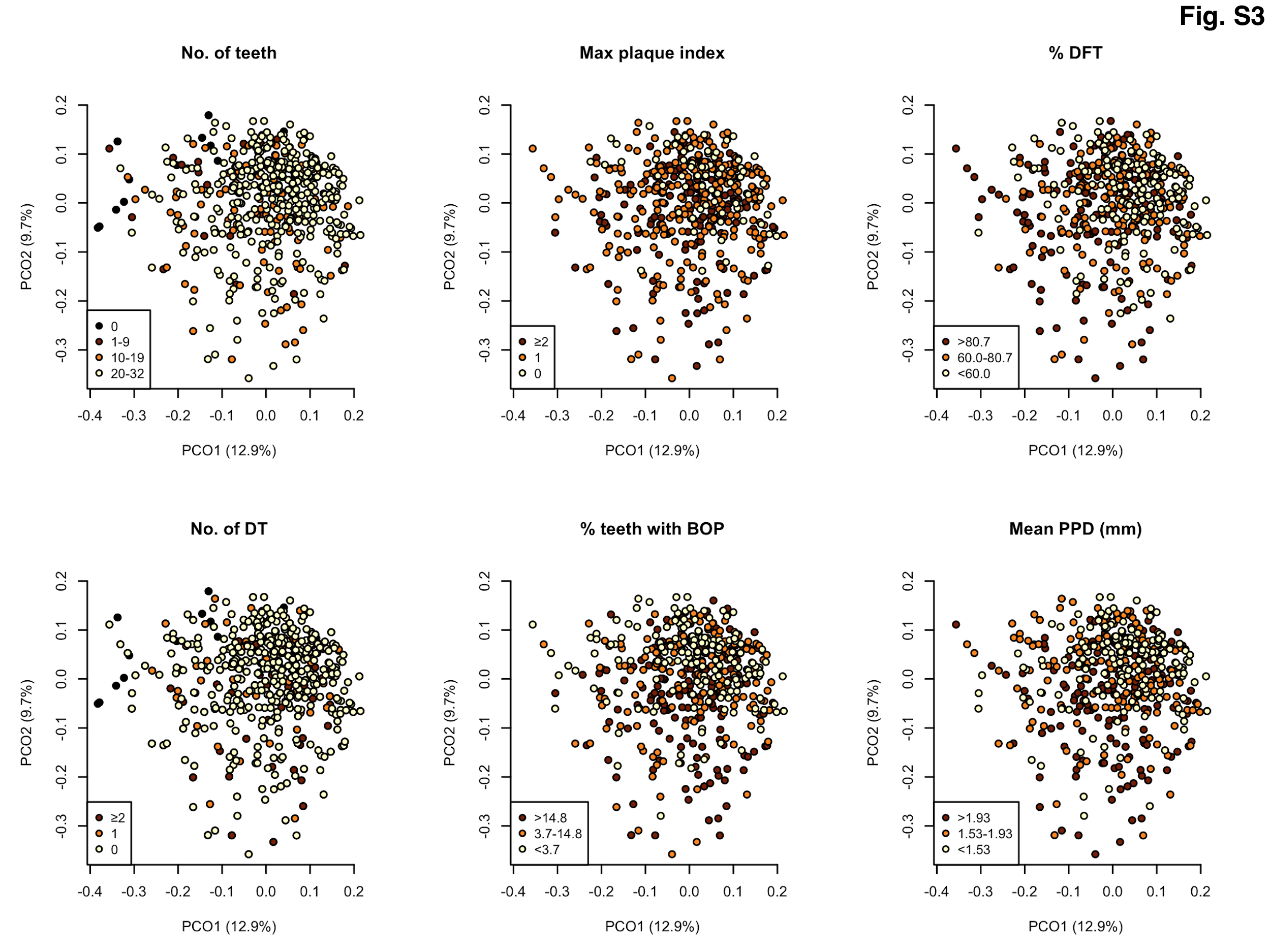

Supplement: FIG S3 [file sph004182614sf3.tif]

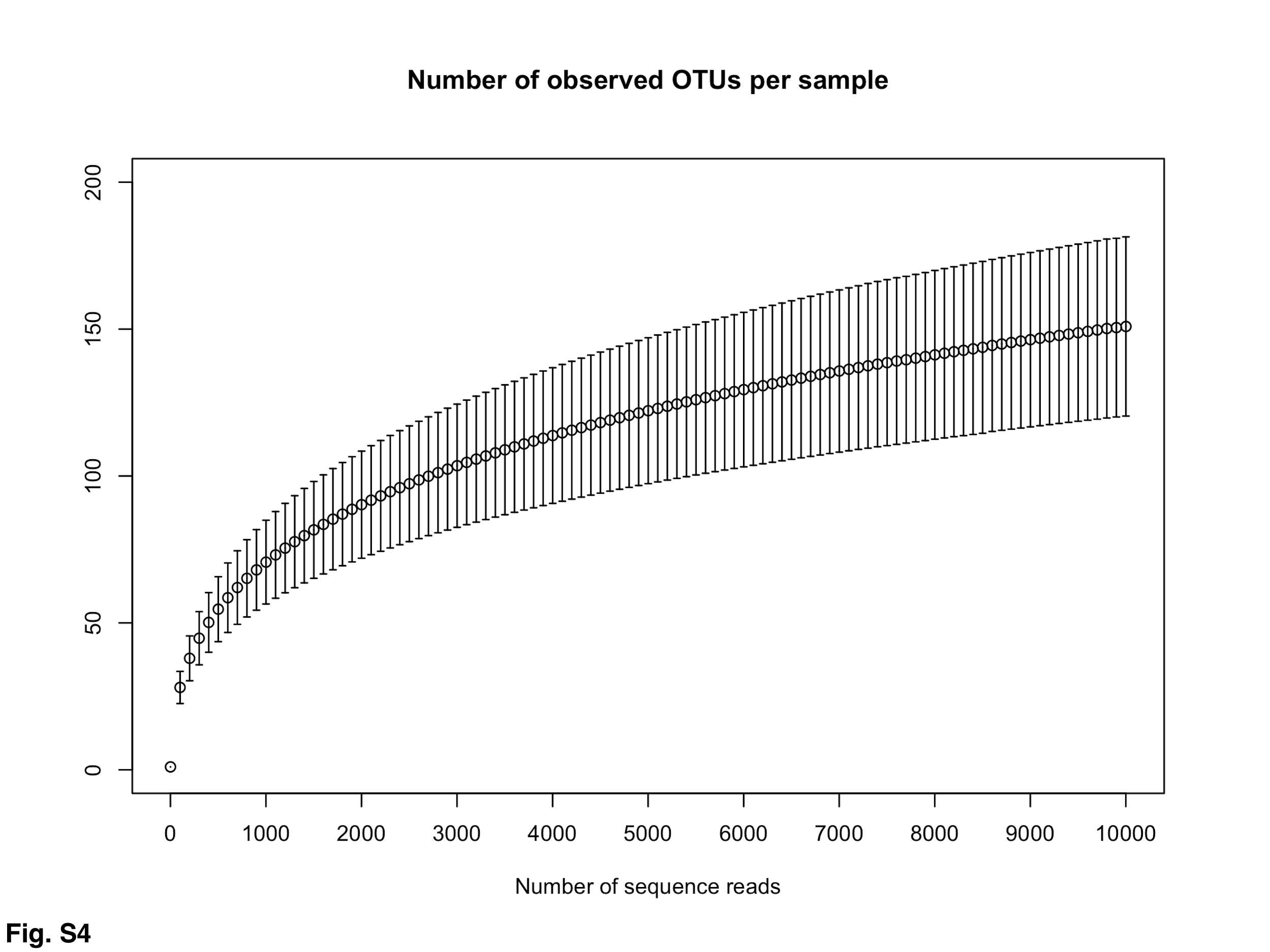

Supplement: FIG S4 [file sph004182614sf4.tif]
