# Supplementary material for: Tongue Microbiota and Oral Health Status in Community-Dwelling Elderly Adults
Source: mSphere. 2018 Aug 15;3(4):e00332-18. doi: 10.1128/mSphere.00332-18 (PMC6094060; doi:10.1128/mSphere.00332-18)
Supplement: TABLE S1 [file sph004182614st1.docx]

**Table S1** Spearman's correlation coefficient between the relative abundances of group I and II commensals and five predominant operational taxonomic units (OTUs) which were not present in both cohabiting groups.

Correlation coefficient

OTU Bacterial species Group I Group II

No. corresponding to each OTU commensals commensals

OTU421 Genus *Neisseria*^a^ -0.35*** 0.05***

OTU22 *Streptococcus* sp. (074) -0.16* 0.10*

OTU23 *Actinomyces graevenitzii* (866) N.S. N.S.

OTU10 *Actinomyces odontolyticus* (701) N.S. N.S.

OTU1 *Rothia mucilaginosa* (681) 0.07** -0.29***

^a^ No blast hit with ≥98.5% identity was found in the Human Oral Microbiome database (HOMD). ****P*< 0.001, ***P*< 0.01, **P*< 0.05. N.S. indicates not significant. Oral taxon IDs in HOMD are given in parentheses following bacterial names.
